# High Resolution Crystal Structure of the Endo-N-Acetyl-β-D-Glucosaminidase Responsible for the Deglycosylation of *Hypocrea jecorina* Cellulases

**DOI:** 10.1371/journal.pone.0040854

**Published:** 2012-07-30

**Authors:** Ingeborg Stals, Saeid Karkehabadi, Steve Kim, Michael Ward, Anita Van Landschoot, Bart Devreese, Mats Sandgren

**Affiliations:** 1 Faculty of Applied Bioscience Engineering, University College Ghent, Ghent, Belgium; 2 Department of Biochemistry and Microbiology, Ghent University, Ghent, Belgium; 3 Department of Molecular Biology, Swedish University of Agricultural Sciences, Uppsala, Sweden; 4 DuPont Industrial Biosciences, Palo Alto, California, United States of America; University of Canterbury, New Zealand

## Abstract

Endo-N-acetyl-β-D-glucosaminidases (ENGases) hydrolyze the glycosidic linkage between the two N-acetylglucosamine units that make up the chitobiose core of N-glycans. The endo-N-acetyl-β-D-glucosaminidases classified into glycoside hydrolase family 18 are small, bacterial proteins with different substrate specificities. Recently two eukaryotic family 18 deglycosylating enzymes have been identified. Here, the expression, purification and the 1.3Å resolution structure of the ENGase (Endo T) from the mesophilic fungus *Hypocrea jecorina* (anamorph *Trichoderma reesei*) are reported. Although the mature protein is C-terminally processed with removal of a 46 amino acid peptide, the protein has a complete (β/α)8 TIM-barrel topology. In the active site, the proton donor (E131) and the residue stabilizing the transition state (D129) in the substrate assisted catalysis mechanism are found in almost identical positions as in the bacterial GH18 ENGases: Endo H, Endo F1, Endo F3, and Endo BT. However, the loops defining the substrate-binding cleft vary greatly from the previously known ENGase structures, and the structures also differ in some of the α-helices forming the barrel. This could reflect the variation in substrate specificity between the five enzymes. This is the first three-dimensional structure of a eukaryotic endo-N-acetyl-β-D-glucosaminidase from glycoside hydrolase family 18. A glycosylation analysis of the cellulases secreted by a *Hypocrea jecorina* Endo T knock-out strain shows the *in vivo* function of the protein. A homology search and phylogenetic analysis show that the two known enzymes and their homologues form a large but separate cluster in subgroup B of the fungal chitinases. Therefore the future use of a uniform nomenclature is proposed.

## Introduction

Endo-N-acetyl-β-D-glucosaminidases (ENGases, EC.3.2.1.96) hydrolyze the β-1,4 linkage in the chitobiose core of N-linked glycans and are thus capable of releasing entire glycans from glycoproteins leaving one N-acetylglucosamine residue on the substrate. This activity is found both in glycoside hydrolase (GH) families 18 and 85 within the family classification of carbohydrate active enzymes [1]. ENGases from GH family 18 (GH18 ENGases) were originally found only in prokaryotes and are evolutionarily related to chitinases. The best-known representatives are Endo H from *Streptomyces plicatus* [2], Endo F1, F2, F3 from *Elizabethkingia meningoseptica* [3] and Endo S from *Streptococcus pyogenes* [4]. The coordinates from another bacterial GH18 ENGase structure (Endo BT from *Bacteroides thetaiotaomicrom*) were deposited in the Protein Data Bank without an associated publication [5]. A new fungal subgroup of ENGases belonging to GH family 18 has recently been discovered. The first biochemically characterized representatives, Endo T from the ascomycete *Hypocrea jecorina* [6], and Endo FV from the basidiomycete *Flammulina velutipes* [7], show low sequence homology with the bacterial ENGases and with the fungal chitinases. However, this deglycosylating activity is widely distributed [7] and several highly homologous proteins or gene products are found among ascomycetes [6]. Both Endo T and Endo FV hydrolyze high-mannose type structures as observed in fungal and yeast glycoproteins, but do not release complex type N-glycans [6], [7]. The *H. jecorina* endo-N-acetyl-β-D-glucosaminidase, Endo T, corresponds with Chi18–20 as described by Karlsson *et al.* in a large phylogenetic study [8] but the enzyme is shown in a previous study not to be involved in chitin degradation [6].

The mature Endo T protein, as purified from the extracellular medium from *H. jecorina* Rut-C30, is N- and C-terminally processed by the removal of 9 and 43 amino acids, respectively [6]. The expression of Endo T is not co-regulated with cellulase production [9], but the enzyme is believed to be responsible for the heterogeneous N-deglycosylation observed for many proteins expressed and secreted by *H. jecorina* [10]–[12].

Proteins have been classified into GH family 18 on the basis of two consensus regions forming the third and fourth β-strand stabilizing the (β/α)8 TIM barrel fold [13]. The GH 18 chitinases and ENGases hydrolyze their substrates with retention of the anomeric configuration [14], [15]. The active site of the family 18 glycoside hydrolases contains two conserved acidic residues at the end of β-strand 4, corresponding to D129 and E131 in Endo T [6]. The glutamic acid has been identified as the proton donor, and the aspartic acid has been assigned a secondary role, stabilizing the intermediate in a substrate-assisted hydrolysis mechanism in which the carbonyl oxygen group of the C2-acetamido of the leaving N-acetyl-D-glucosamine (GlcNAc) acts as the nucleophile [16], [17]. Currently there are 49 GH family 18 protein structures deposited at the protein data bank (PDB), among which only 4 represent bacterial ENGases (Endo H [PDB accession code 1EDT], Endo F1 [2EBN], Endo F3 [1EOM] and Endo BT [3POH]).

The structure of Endo H, Endo F1 and several family 18 chitinases have a typical β-hairpin loop in the loop connecting the β-strand and the α-helix in unit 2 of the TIM barrel, which in previous studies has been shown to be important for substrate recognition [13], [18]–[20]. In the structure of Endo F3 there are two 1.5 turn α-helices in the loops connecting the β-strand and α-helix in units 2 and 3 [21]. The structure of Endo F3 in complex with an octasaccharide biantennary oligosaccharide shows that only residues from the Man-α(1–3)(Man-α(1–6))-Man-β(1–4)-GlcNAc core, shared by all N-linked oligosaccharides, make direct contact with the protein [21].

In this study, the 1.3 Å crystal structure is described as the first fungal representative in GH family 18 with endo-N-acetyl-β-D-glucosaminidase activity, Endo T from *H. jecorina*. The structure is compared with the previously known bacterial family 18 structures with the same activity. Evidence is given that the Endo T enzyme is indeed responsible for the occurrence of single N-acetylglucosamine residues on *H. jecorina* (hemi-)cellulases by glyco-analysis of the secretome of the knock-out strain. Since the fungal ENGases make up a separate phylogenetic subgroup among the GH18 proteins and the activity has been biochemically proven for two members [6], [7] we here propose to use a nomenclature [22] for these endo-N-acetyl-β-D-glucosaminidases within GH family 18 that clearly differentiates these enzymes from the chitinases within the family; e.g. HjEng18A for *Hypocrea jecorina* Endo T and FvEng18A for *Flammulina velutipes* Endo FV.

## Results

### Glyco-analysis of the secretome of the knock-out strain

**Figure 1:**
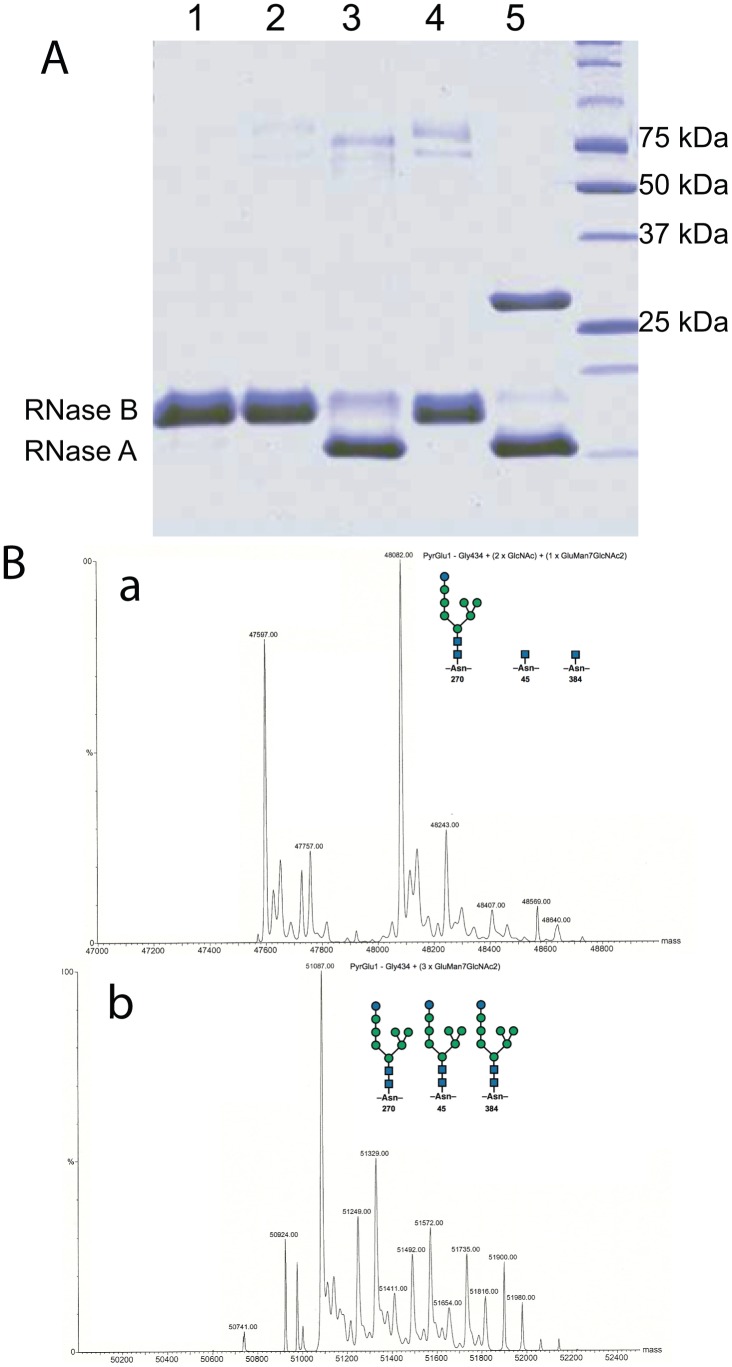
Detection of deglycosylating activity. Figure 1A; SDS-PAGE analysis showing deglycosylating activity: RNAse B band shift analysis with negative control (lane 1), medium of Endo T knock-out transformant 4 (lane 2), medium of RL-P37 wild type strain (lane 3), medium of Endo T knock-out transformant 10 (lane 4) and positive control with purified Endo T (lane 5). Figure 1B; ESI-MS spectrum of the purified catalytic domain from Cel7A secreted by the wild type (a) and Endo T knock-out strain (b). The catalytic core of this protein carries three N-glycans found at Asn45, Asn270 and Asn 384 [10], [57] ^.^ The core protein originating from the wild type strain (a) has been partially deglycosylated due to endoglucosaminidase activity in the medium while the protein from the knock-out strain (b) still contains its three N-glycans.

The wild type and Endo T knock-out strain of *Hypocrea jecorina* RL-P37 were grown in corn steep liquor enriched medium to promote post-secretorial trimming of the glycans, as described before [23]. Band shift analysis with the glycoprotein RNase B was used to show the presence or absence of deglycosylating activity in the media. Only with medium from the wild type RL-P37 strain, there was conversion of the RNase B substrate into RNase A (Fig. 1A, lane 3) while the media from the knock-out transformants (lanes 2 and 4) did not show any deglycosylating activity. Staining of the proteins present in the media also showed cellulases with a higher molecular weight in the Endo T knock-out strain compared to the wild type strain (Fig. 1A, lanes 3 and 4). The presence of the N-glycans was further proven by ESI-MS analysis of the catalytic domain of *H. jecorina* Cel7A: the core protein originating from the wild type strain has been partially deglycosylated due to ENGase activity in the medium while the cellulase from the knock-out strain still contains its three N-glycans (Fig 1B).

### Protein expression and characterization

The Endo T protein (CAZ16624.1, 359 amino acids) was overexpressed in a *H. jecorina* production strain deleted for the four main cellulases genes under the control of the *H. jecorina* cel7a promoter. Upon lactose induction in shaker flask culture, total protein expression levels of 1.2 g/L were obtained. It was confirmed by band shift analysis that the expressed Endo T protein was highly active (data not shown). The Endo T protein was post-translationally processed: SDS-PAGE analysis revealed a major protein band of 32 kDa. The N- and C-terminal sequences of the purified protein were determined as AEPTDL and GL, respectively. The loss of signal by C-terminal sequencing was probably due to a penultimate Pro residue. The ESI-MS spectrum showed a single species of 31 755 Da (data not shown). The mass of the protein sequence (A1-L287) and two GlcNAc residues (due to auto-deglycosylation) perfectly accounts for this experimentally determined molecular mass. These results suggest that the C-terminus has been processed at a position three residues further upstream compared with the previously characterized protein isolated from the Rut-C30 strain [6]. Although the 37 kDa protein form was never observed in the *H. jecorina* medium, we could not show unambiguously if the proteolytic processing happened intra- or extracellular.

### Crystallization, structure solution and quality of the final model

Crystals of Endo T were grown by the vapor diffusion crystallization technique and could be grown in various crystallization solutions. After testing the initial crystals at a synchrotron source, it was shown that a crystallization solution containing zinc acetate, sodium acetate and PEG3350 yielded the best diffracting crystals. Zinc might have an impact on the crystal packing since crystallization solutions without zinc gave rise to poorer X-ray diffraction data. Since zinc was included in the crystallization conditions, it seemed obvious to make an attempt to use zinc as the source of anomalous scattering for structure determination.

The Endo T structure was indeed solved by Multiple Anomalous Dispersion (MAD) techniques to a resolution of 2.15 Å using a zinc MAD dataset collected on beam-line ID911:3 at the Swedish synchrotron source MAX-Lab in Lund. Subsequently, a 1.3 Å resolution native Endo T data set was collected on a different crystal. Further statistics for data collection and processing are presented in Table 1. The final Endo T structure model, based on the 1.3 Å high resolution native dataset, contains 2237 non-hydrogen atoms belonging to one protein molecule consisting of 283 amino acid residues, two N-acetylglucosamine residues, seven zinc ions and 434 water molecules and 3 acetate molecules. The deposited Endo T structure model has a crystallographic R and an R-free value of 18.4 and 20.1 %, respectively. Other structure model refinement statistics are listed in Table 2. In the final 2mF_o_-DF_c_ σA weighted [24] electron density map, the electron density is continuous for all main-chain atoms of the protein from D5 to G286. These amino acids correspond to D31 and G312 in the GeneBank deposited amino acid sequence (CAZ16624.1). In the Ramachandran plot [25] there were no outliers by the stringent core definition given by Kleywegt and Jones [26], and other geometric parameters only show small deviations from ideal values.

**Table 1:** Data collection and processing statistics.

Endo-T dataset	Native	Zn peak	Zn inflection	Zn remote
Beamline^a^	I911–1	I911–3	I911–3	I911–3
Wavelength (Å)	1.03700	1.28101	1.28199	1.27200
No. of images	195	180	180	180
Oscillation range (^o^)	1.0°	1.0°	1.0°	1.0°
Space group	P2_1_	P2_1_	P2_1_	P2_1_
Cell parameters				
a = (Å)	35.4	35.4	35.4	35.4
b = (Å)	63.9	63.8	63.8	63.8
c = (Å)	59.4	59.4	59.4	59.4
β = (°)	101.0	100.8	100.8	100.8
Resolution range (Å)	19.4–1.3	18–2.15	18–2.15	18–2.15
Completeness (%)^b^	99.5 (95.6)	100 (100)	100 (100)	100 (100)
Resolution range outer shell	1.33–1.30			
No. of observed reflections	246096			
No. of unique reflections	63365			
Average multiplicity	3.9 (3.6)			
R _merge_ (%)^c^	7.0 (20.0)			
I/σ(I)	12.3 (5.5)			
Phasing statistics				
Resolution cutoff Å		18–2.15		
Number of zinc sites found		7		
Overall FOM		0.52		
Score after phasing		0.47		
Map corr. coef.		0.72		

a Beamline at MAX-lab, Lund, Sweden.

b Numbers in parentheses are for the highest resolution bins.

c R_merge_ = Σ_hkl_ Σ_i_|I – < I >|/Σ_hkl_ Σ_i_ | I |.

**Table 2:** Refinement and final structure model statistics.

PDB access code	4AC1
Resolution used in refinement (Å)	20–1.3
Reflections in: working & test set	60140 & 3206
R^a^ & R_free_ factor (%)	18.4 & 20.2
Protein molecules in AU	1
Residues in protein	283
Protein atoms	2720
Waters	440
Residues with double conformations	12
N-glycosylation (GlcNAc)	2
Average atomic B-factor (Å^2^):	10.5
RMSD bond lengths from ideal (Å)	0.008
RMSD bond angles from ideal (°)	1.241
Ramachandran plot statistics (%)	
Most favorable regions	97.9
Allowed regions	2.1
Disallowed regions	0.0

a R = Σ | |F_o_|– |Fc| |/Σ |F_o_|; the final R-factor is given.

### Protein fold and description of the structure

**Figure 2:**
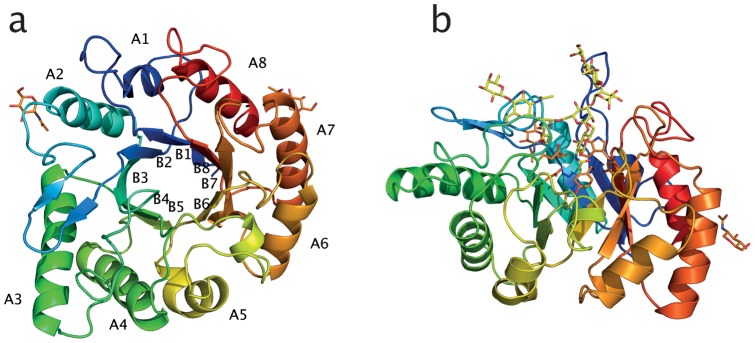
Cartoon representation of the crystal structure of *H. jecorina* Endo T, top view (a), and side view (b). The Endo T structure is rainbow colored according to residue number, starting with blue at the N-terminus and ending with red at the C-terminus. In figure (a) the nomenclature of α-helices 1 to 8 and β-strands 1 to 8 building up the (β/α)_8_-TIM barrel is indicated. In figure (b) the octasaccharide found bound in the ligand complex structure of *E. meningoseptica* Endo F3 (PDB ID 1EOM) has been modeled in the active site of Endo T to indicate its position in the enzyme. Single GlcNAc residues at positions N70 and N240 (due to auto-deglycosylation) are shown in stick format and colored orange. Figure prepared with the program PyMol [52].

**Figure 3:**
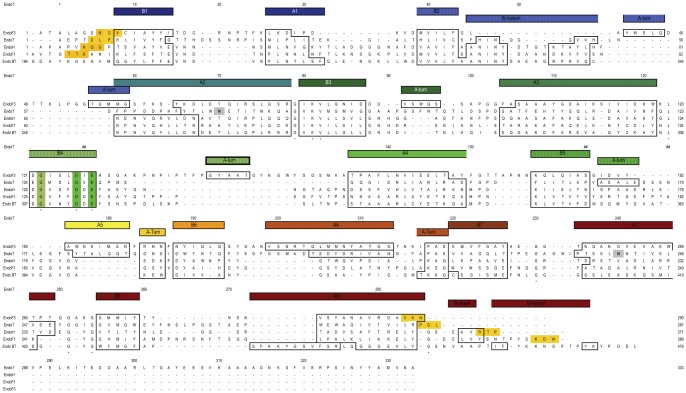
Structure based sequence alignment of the four GH family 18 proteins that possess endo-N-acetyl-β-d-glucosaminidase type activity, and with known three-dimensional structure. The important active site residues are highlighted with a green background. The secondary structure assignment (boxes), indicated on top of the sequence alignment, is rainbow colored according to the residue number, starting with blue at the N-terminus and ending with red at the C-terminus. The shown aligned sequences are (from the top); *Elizabethkingia meningoseptica* Endo F3 (PDB ID 1EOM, Uniprot access code P36913), *Hypocrea jecorina* EndoT (PDB ID 4AC1, Uniprot access code C4RA89); *Streptomyces plicatus* Endo H (PDB ID 1EDT, Uniprot access code P11797.1); *Elizabethkingia meningoseptica* Endo F1 (PDB ID 2EBN, Uniprot access code P36911.1), and *Bacteroides thetaiotaomicron* Endo BT (PDB ID 3POH, Uniprot access code Q8A0N4). The glycosylated Asn in the sequons of *H. jecorina* EndoT are shaded grey. Yellow shaded amino acids are the C-terminal residues observed in the respective crystal structures.

Although 46 amino acids are missing at the C-terminus of the protein, the overall fold of Endo T is a complete (β/α)_8_-TIM barrel (Fig. 2 and 3). The core of the TIM barrel structure consists of a twisted β-sheet, which is composed of eight parallel β-strands that are surrounded, and connected, by eight α-helices located on the surface of the molecule. Figures 2 and 3 illustrate the nomenclature of the α-helices and β-strands. As suggested by Hennig *et al*. [27] the connecting loops will, in the following text, be referred to as βxαx for loops from β-strand x to α-helix x and αxβx+1 for loops from α-helix x to β-strand x+1, respectively. In (β/α)_8_ enzymes, the active site is located in a cavity at the C-terminal end of the parallel β-barrel. The βxαx loops on the top of the barrel have the greatest variation and define the substrate binding site of the enzyme, as also for Endo T (as shown in Fig. 2b). The αxβx+1 loops located on the opposite side of the barrel, only demonstrate some minor variations in length and conformation.

**Figure 4:**
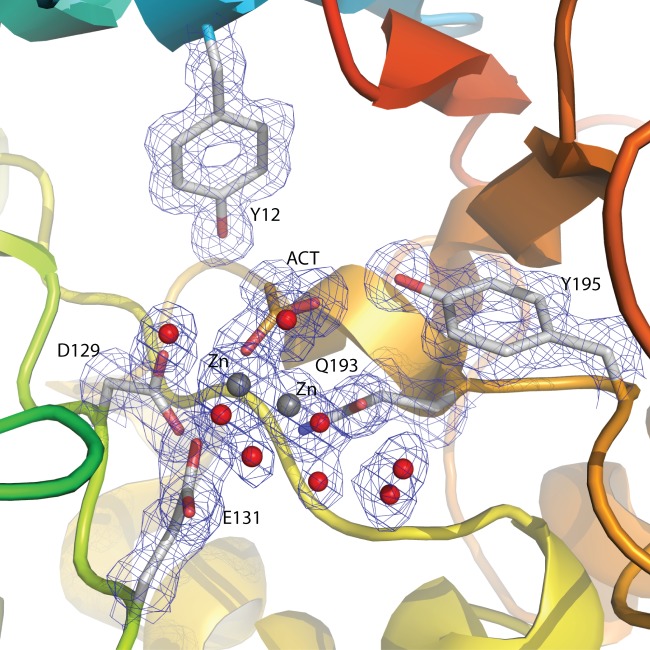
Electron density of the zinc atom bound in the active site of *H. jecorina* Endo T structure. The zinc atom, modeled in dual conformation, is shown in grey spheres and the surrounding water molecules that are involved in the coordination spheres of zinc are shown in red spheres. Two water molecules coordinating the zinc atom in the active site have also been modeled in dual confirmation. The displayed maximum likelihood/_σA_ weighted 2F_obs_−F_calc_ electron density map, contoured at 1.0 σ level (0.38 e/Å^3^), is shown in greyish-blue. Figure prepared with the program PyMol [52].

The Endo T structure is composed of ten strands and 11 helices, and the structure has approximate dimensions of 45 Å×34 Å×57 Å. The two GH family 18 consensus regions, corresponding to the amino acids forming the third and fourth strand of the barrel, and the two carboxylic acids (D129 and E131), playing a key catalytic role, are structurally highly conserved among the five ENGases in family 18 (shaded in Fig. 3). Seven zinc atoms have been modeled in the structure model of Endo T, six of which are located at the surface of the molecule. The seventh zinc atom is bound deep in the active site of the enzyme in a pocket formed by the catalytic residues (Fig. 4 and S1). This zinc atom has dual conformations in the structure model and it is coordinated by the two catalytic residues. Several water molecules are also bound in the active site of the enzyme. The Endo T structure also shows two single GlcNAc residues bound at two of the predicted N-glycosylation sites of the enzyme, N70 and N240 respectively (Fig. 2).

### Comparative analysis of the structure of Endo T

Four representative ENGase structures from GH family 18 have previously been reported: Endo H [PDB accession code 1EDT] from *Streptomyces plicatus*, Endo F1 [2EBN], Endo F3 [1EOK] from *Elizabethkingia meningoseptica* and Endo BT [3POH] from *Bacteroides thetaiotaomicron*. A superposition of the Endo T structure with those of Endo H, Endo F1, Endo F3 and Endo BT using the program LSQMAN [28], gives Root Mean Square Deviation (RMSD) values of 1.81 Å, 1.93 Å, 2.12 Å and 1.89 Å (for 152, 156, 187 and 149 C-α atom pairs), respectively. Rao *et al*. have shown that the structures of Endo H and Endo F1 are very similar [19]. The C-terminal domain of the recently released Endo BT structure also has a high similarity as observed in our structure based sequence alignment (Fig. 3). Endo H, Endo F1 and Endo T enzymes have nearly identical substrate specificities hydrolyzing high mannose type N-glycans [3], [6]. No activity study has yet been reported for Endo BT. Endo F3 has a different substrate specificity compared with the other three enzymes, accepting complex bi- and tri-antennary type N-glycans [29]. This was rationalized with the crystal structure of Endo F3 in complex with its bi-antennary octasaccharide product (PDB accession code 1EOM), as described by Waddling *et al*. [21].

**Figure 5:**
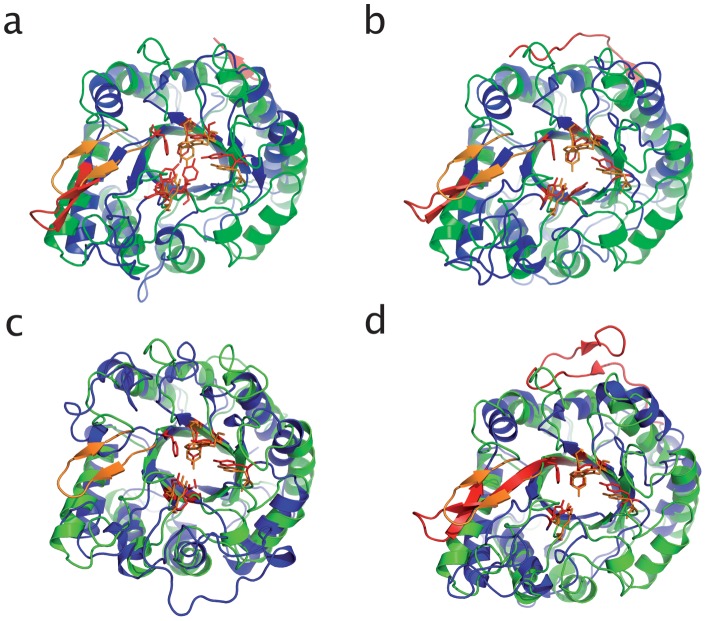
Cartoon representation overlay of the superimposed structures of; (a) *H. jecorina* Endo T, colored in green, and *S. plicatus* Endo H (PDB ID 1EDT), colored in blue. The hairpin loop of Endo T, colored in gold, is shorter than the corresponding loop of Endo H, colored in red; (b) *H. jecorina* Endo T, colored in green, and *E. meningoseptica* Endo F1 (PDB ID 2EBN), colored in blue. The hairpin loop of Endo T, colored in gold, is shorter than the corresponding loop of Endo F1, colored in red. The loop at the C-terminal of Endo F1 is also colored in red; (c) *H. jecorina* Endo T, colored in green, and *E. meningoseptica* Endo F3 (PDB ID 1EOM), colored in red. The hairpin loop is completely missing in the structure of *E. meningoseptica* Endo F3; (d) *H. jecorina* Endo T, colored in green, and *B. thetaiotaomicron* Endo BT (PDB ID 3POH). Figure prepared with the program PyMol [52].

Visual inspection of the superimposed structures reveals that most of the secondary elements are situated at the same position in the five family 18 ENGase structures (Fig. 5). As expected, the greatest variations among the structures are found in the length of the βxαx loops on the top of the barrel forming the substrate binding cleft. Endo T shows overall the highest structural similarity with Endo F3. Both Endo T and Endo F3 have a complete (β/α)_8_-barrel, while in the Endo H and Endo F1 structure the α-helices α5 and α6 are missing. Several other differences were observed when comparing the structures, as described below and represented in figure 3.

For instance, the β1α1 loop is 4 to 8 residues longer in the Endo F3 and Endo T structures compared to the corresponding loop in Endo H (Endo F1 and Endo BT) (Fig. 3). In previous structural comparisons, the last residue of β strand 2 which is a conserved phenylalanine (F44 in Endo H) has been proposed to be important for substrate recognition [27], [30]. In the Endo T structure however, this corresponds to a cysteine (C43). This residue forms a *cis* peptide bond with the next residue which initiates a β hairpin (formed by two short β sheets H46-N48 and V52-H54) found in the β2α2 loop (colored in Fig. 5). This hairpin is a common feature in family 18 proteins. If we compare this hairpin loop with the one in Endo H, Endo F1 and Endo BT, this loop is found in a similar position but is four residues shorter (red in Fig. 5a, b and d). The hairpin is completely missing in the structure of Endo F3 (Fig. 5c) as reported before [21].

The β3α3 loop is relatively long in all compared structures and is located next to the catalytic acids. This loop shows a lot of structural variation among the five structures: Endo F3 has an α-turn in this loop (Fig. 3), Endo H, Endo F1 and Endo BT have a much shorter loop, while in Endo T this loop is highly structured and actively takes part in building up the active site of the enzyme. Remarkably, the β4α4 loop in Endo H adopts a similar configuration as the β3α3 loop in Endo T and thus seems to compensate for its shorter β3α3 loop (Fig. 5a). In fact, several loops in this region in Endo H are shifted one secondary element, which could be explained by the missing helices α5 and α6 in this structure. The β4α4 loop is very short in the Endo T structure while Endo F3 has an extensive 30 amino acid long loop containing a short α-turn. The opposite occurs with the β5α5 loop with the Endo T protein now having the longest loop with an α-turn. Both loops are positioned so that they are likely to interact with the protein part of the glycoprotein substrate. Part of the elaborated β4α4 loop in Endo F3 (N149–S155) has the same position of the β5α5 loop in Endo T (S168–S172).

**Figure 6:**
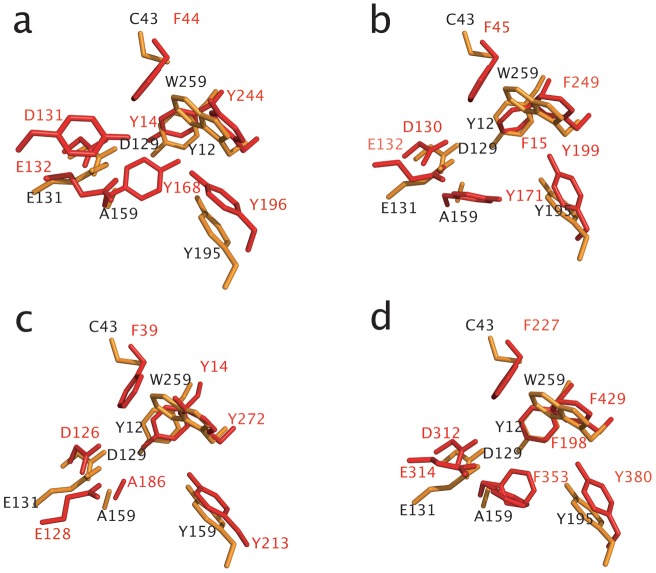
Overlay of the superimposed active site residues of; (a) *H. jecorina* Endo T, and *S. plicatus* Endo H (PDB ID 1EDT); (b) *H. jecorina* Endo T and *E. meningoseptica* Endo F1 (PDB ID 2EBN), (c) *H. jecorina* Endo T, and *E. meningoseptica* Endo F3 (PDB ID 1EOM), and (d) *H. jecorina* Endo T, and *B. thetaiotaomicron* Endo BT (PDB ID 3POH). The active site residues of Endo T are depicted in orange and those of Endo F1, Endo H, and Endo F3 in red. Figure prepared with the program PyMol [52].

In all GH family 18 structures compared here, there is a conserved tyrosine residue at the beginning of the β6α6 loop (Y195 in Endo T, Fig. 6). The β6α6 loop is slightly longer in Endo T compared to the other enzymes, but this comparison is hampered by to the missing α6-helix in Endo H, Endo F1 and Endo BT. Both the β7α7 and β8α8 loops are again longer in Endo T compared to the other three structures, and could take part in substrate recognition. The α8-helix is the last secondary structure element of the TIM barrel. In the structure of Endo BT, this helix is broken and much longer than in the other four structures.

**Figure 7:**
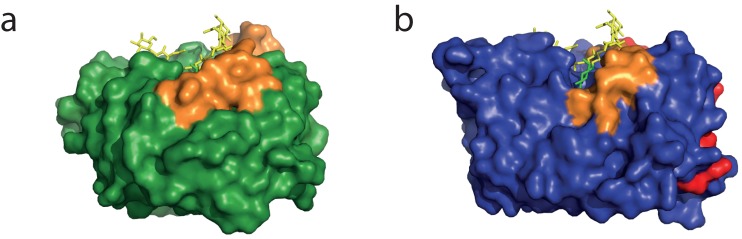
Structure model surface representations of (a) the *H. jecorina* Endo T structure, colored in green and (b) *Streptomyces plicatus* Endo F1 (PDB ID 2EBN), colored in blue. The extended β1α1, β6α6 and β7α7 loops of *H. jecorina* EndoT are colored in gold. The C-terminal peptide in Endo F1 is colored in red. The octasaccharide found bound in the ligand complex structure of *E. meningoseptica* Endo F3 (PDB ID 1EOM) has been modeled in the active site of Endo T. Figure prepared with the program PyMol [52].

The 46 amino acid peptide following the α8-helix in Endo T is absent in the presented structure due to proteolytic cleavage as shown by mass spectrometry. The bacterial ENGase structures from Endo H, Endo F1 and Endo BT have respectively 8, 15, and 25 amino acids following the barrel. These form a structured loop at the C-terminus that folds back on the barrel. The loops of Endo F1 and Endo BT stretch towards the active site (shown in red in Fig. 5 and Fig. 7). Although the sequence similarity is low (Fig. 3), the structures superimpose well, and in all cases, several hydrogen bonds and hydrophobic interactions keep this C-terminal loop in its position (data not shown).

### Active site and a possible binding site for the oligosaccharide

In all five GH18 ENGase structures compared in this study, the two carboxylic acids, involved in the substrate-assisted catalytic mechanism, are found at similar positions at the end of β-strand 4 (Fig. 6). These two catalytic residues (D129 and E131 in Endo T) are surrounded by residues that all can be important for substrate binding. In Endo F3, (Fig. 6c) the tyrosine residue Y213 interacts with the acetyl group of the second GlcNAc residue of the N-glycan. In Endo T, the conserved tyrosine Y195 is present in an almost identical position and a zinc atom is found bound at the exact same position where the reducing GlcNAc molecule is found in the Endo F3 structure. Another aromatic residue in Endo T (Y12) is found in similar positions in all five structures. This tyrosine has been proposed by Fujita *et al*. to be important for activity based on mutagenesis studies [30]. A third conserved aromatic residue in Endo F3 (W259 in Endo T) forms a hydrophobic platform forming stacking interactions with the GlcNAc residue in the −1 subsite.

The overall comparison of the five structures shows that Endo T possesses a slightly deeper and narrower substrate-binding cleft than the other enzymes. For instance, three longer loops in the Endo T structure, β1α1, β6α6 and β7α7 (Fig. 7), are pointing directly to the center of the barrel, hereby forming a more complex substrate binding platform compared with the other four structures. We can only speculate if this would alter the substrate affinity or specificity of the enzyme. Apart from the aromatic residues Y12, Y195 and W259 in the Endo T structure, already discussed above, there are additional aromatic residues in the vicinity of the substrate in the other structures that are absent in the Endo T structure. For instance, F44 and Y168 in Endo H (Fig. 3 and 6) are exchanged by C43 and A159, respectively, in Endo T. For a third aromatic residue in Endo H, Y133, adjacent to the proton donor, E131, no equal amino acid exists in the Endo T structure.

### Phylogenetic classification

**Figure 8:**
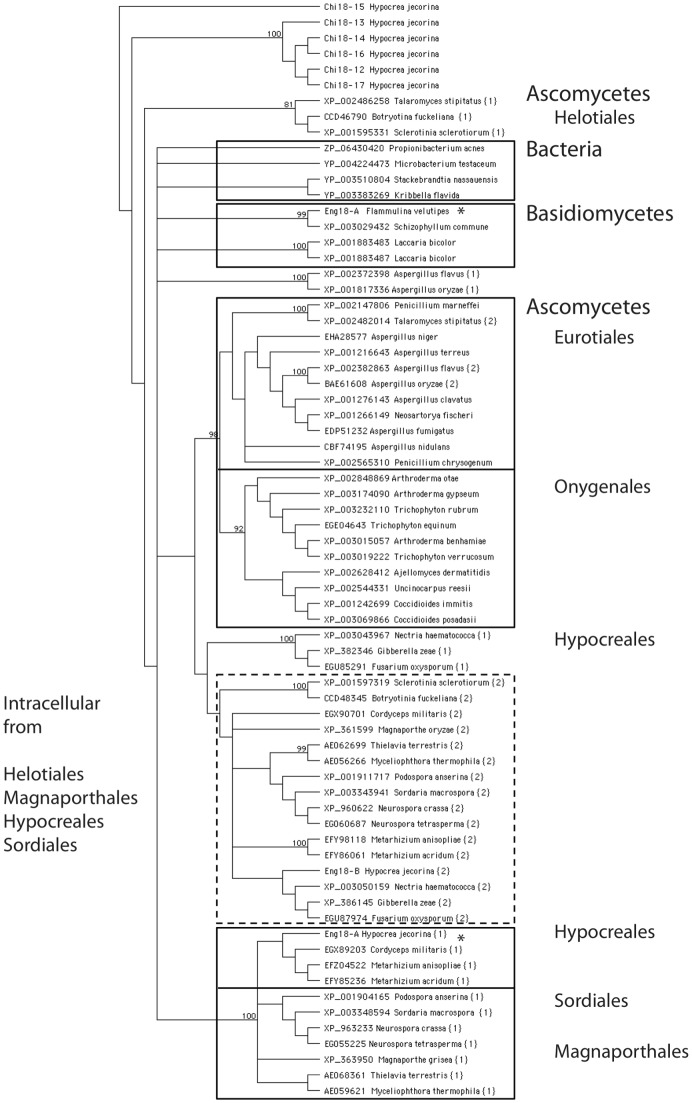
Phylogenetic tree of GH family 18 ENGases, group B. The phylogenetic tree is based on an amino acid sequence alignment (CLUSTALX) and was constructed by neighbour joining. Bootstrap values are based on 1000 replications and nodes that have bootstrap support above 70% are indicated with the percentage. The tree is rooted with fungal GH family 18 chitinases belonging to the same subgroup. Previously characterized ENGases are indicated with an asterisk*. Boxes indicate proteins belonging to micro-organisms of the same order as described in the text.

The two *H. jecorina* endo-N-acetyl-β-D-glucosaminidase genes Chi18–19 and Chi18–20 (Endo T) were previously shown to cluster in the B–V subgroup of fungal GH18 genes [31]. In the current analysis, a rooted phylogenetic tree was constructed that included the three fungal ENGases (Endo T, Endo FV and Chi18–19) and the first 100 orthologues. Characterized bacterial ENGases were excluded from the analysis, as they could not be unambiguously aligned. GH18 subgroup B-I/B-II *H. jecorina* chitinases (except Chi18–18) [8] were included and used to root the tree. As shown in figure 8, all included ENGases form one phylogenetic cluster in subgroup B of fungal GH18 proteins, which correspond to group B–V in previous studies [8], [31]. This suggests that fungal GH18 ENGases evolved once from an ancestral GH18 enzyme with chitinolytic activity. Since the activity has been biochemically proven for two fungal B–V members [6], [7] and these enzymes are often wrongly annotated as chitinases, we suggest to use a systematic and more uniform nomenclature. We follow the proposal of Henrissat [22] to include the glycoside hydrolase family number after the three-letter code of the gene (eng). Eng was chosen because it is the abbreviation of endo-N-acetyl-β-D-glucosaminidase and it was already used in the first reports describing this activity [32]. Moreover, the name is used throughout the literature describing intra- and extracellular enzyme activities belonging both to GH family 18 and 85 [33]. In this way, Endo T (protein ID 65162 or Chi18–20) and Chi18–19 (protein ID 121355) would be named *H. jecorina* Eng18A and Eng18B respectively.

A more detailed analysis of the ENGase orthologues (Fig. 8) shows that the majority of the proteins belong to the Ascomycetes while only four members are found among the Basidiomycetes and four among the bacteria. These three groups are well separated in the phylogenetic tree. The proteins from the Basidiomycetes lack a secretion signal and include the biochemically characterized enzyme FvEng18A (Endo FV) from *Flammulina velutipes* [7], two GH18 proteins from *Laccaria bicolor* and one from *Schizopyllum commune*. The bacterial ENGases are restricted to the order of the Actinomycetales and originate from *Propionibacterium acnes*, *Stackebrandtia nassauensis*, *Kribella flavida* and *Microbacterium testaceum*. The proteins in the Ascomycetes are more diverse and belong to different orders. A separate cluster (including HjEng18B) exists where proteins from both the order of the Helotiales, the Magnaporthales, the Hypocreales and the Sordiales, are present (indicated by the dashed box in Fig. 8). These enzymes do not contain a secretion signal and are probably residing in the cell. Interestingly, all these organisms have a second gene product. These proteins are clustered with members from the same order (boxed in Fig. 8). The characterized HjEng18A (Endo T) is grouped with its closest homologues within the Hypocreales, the Magnaporthales and the Sordiales. The majority contain a signal peptide or a Kex2-like cleavage site and these proteins are therefore most likely secreted in the extracellular environment. Several proteins are also present originating from the orders of the Onygenales and the Eurotiales (e.g. *Aspergillus* proteins) but neither of them has a counterpart in the HjEng18B (Chi18–19) subgroup. These latter representatives are again characterized by the absence of a secretion signal.

## Discussion

The mannosyl glycoprotein endo-N-acetyl-β-D-glucosaminidase (Endo T, HjEng18A) is shown to be responsible for the microheterogeneity observed for *Hypocrea jecorina* cellulases and hemicellulases. The enzyme was crystallized and the structure was determined to a resolution of 1.3 Å. Although the mature Endo T protein lacks 46 amino acids at the C-terminus of the predicted protein, the structure forms a complete (β/α)_8_ TIM barrel, a fold that is shared among all glycoside hydrolase family 18 proteins with known structure. The sequences of the four bacterial GH family 18 endo-β-*N*-acetylglucosaminidases with known structure have very low sequence identity with the fungal HjEng18A but the cores of these structures superimpose very well. Only the βxαx loops connecting the β-strands and α-helices forming the core of the TIM-barrel differ significantly among the structures, presumably for the accommodation of different substrates.

HjEng18A clusters with the characterized FvEng18A (Endo FV) protein in a separate phylogenetic group of cluster B of the GH18 proteins as suggested before by Karlsson et al. [8]. Clear proof was given in previous reports that these enzymes are important for protein deglycosylation and not for chitin degradation [6], [7]. Glyco-analysis of the secretome of the *H. jecorina* RL-P37 knock-out strain further strengthens this. Probably the highly homologous proteins present in the same cluster, are ENGases as well. Fungi from the order of the Sordiales, the Hypocreales and the Magnaporthales all have an orthologous gene (HjEng18B for *Hypocrea jecorina*). These enzymes could, in analogy with plant GH family 85 ENGases [34], [35], be involved in the endoplasmic-reticulum-associated protein degradation (ERAD) pathway since they have no signal sequence. Moreover, for *H. jecorina*, the genome does not contain other deglycosylating enzymes (such as PNGase F-type or GH85 ENGases activity) [36] that could play this important role in the cell. The two *H. jecorina* proteins (HjEng18A and HjEng18B) complement the list of eighteen *H. jecorina* chitinases from GH family 18 (HjChi18–1 to HjChi18–18) described by Seidl *et al*. [37], [38].

Future HjEng18A characterization work will be focused on determination of a structure of the enzyme in complex with its natural substrate, and the structure of the intact form of the protein. Further structural information could point towards the function of the proteolytic cleavage at the C-terminus of the HjEng18A enzyme.

## Materials and Methods

### Deletion of the EndoT gene in *Hypocrea jecorina* RL-P37

Flanking regions from the *H. jecorina* EndoT locus were amplified by PCR. The 5′ flanking region was 1.9 Kb and the 3′ flanking region was 1.7 Kb in length. These were inserted into a cloning vector and a mutant form of the *H. jecorina* acetolactate synthase gene conferring resistance to chlorimuron ethyl (WO 2008/039370) was inserted between them to create the deletion cassette. This deletion cassette was subsequently excised from the vector by restriction enzyme digestion and was purified by preparative agarose gel electrophoresis.

*H. jecorina* strain RL-P37 was transformed with the deletion cassette using PEG-mediated transformation of protoplasts [39]. The transformants were selected on Vogel's medium with glucose and 200 ppm chlorimuron ethyl. Transformants were cultured in liquid medium and culture supernatants were analyzed by SDS gel electrophoresis. Two transformants displayed an upward shift in mobility of most of the protein bands on the gel as expected if the proteins had a higher extent of glycosylation. Chromosomal DNA was isolated from these two strains as well as the parent RL-P37 strain of *H. jecorina*. PCR analyses confirmed the expected integration of the deletion cassette at the *endoT* locus and loss of the *endoT* open reading frame. The deleted transformants were subjected to two successive rounds of purification by isolation of colonies from single spores.

The knock-out strain was precultivated at 28°C for 3 days in glucose (20 g/L) containing minimal medium (50 ml) and then induced for cellulose production with lactose (20 g/L) in rich medium (300 ml) for 3 days. The growth medium contained per L: 5 g (NH_4_)_2_SO_4_; 0.6 g CaCl_2_; 0.6 g MgSO_4_; 15 g KH_2_PO_4_; 15×10^−4^ g MnSO_4_; 50×10^−4^ g FeSO_4_ 7H_2_O; 20×10^−4^ g CoCl_2_; and 15×10^−4^ g ZnSO_4_. The rich medium was enriched with 4.2% corn steep liquor (Sigma). The extracellular medium was harvested and concentrated by diafiltration (Amicon stirring cell) using a polyethersulfon membrane with a 3 kDa cut-off (Millipore).

### Expression vector construction

For over-expression of Endo T in *H. jecorina* an integrative expression vector, pTrex3g, was used ((WO/2005/001036) Novel *Trichoderma* genes). This vector is based on the *E. coli* plasmid pSL1180 (Pharmacia Inc., Piscataway, NJ). It was designed as a Gateway destination vector [40] to allow insertion using Gateway technology (Invitrogen) of any gene or part thereof downstream of the strong *H. jecorina* cel7a promoter. The plasmid also contains the *Aspergillus nidulans* amdS gene, with its native promoter and terminator, as selectable marker for transformation of *H. jecorina*.

The ORF of the Endo T gene was amplified from *H. jecorina* genomic DNA by PCR using the primers Endo Ta (CACCATGAAGGCGTCCGTCTACTTG) and Endo Tb (CCCTTAAGCATTCACCATAGC) and inserted into pENTR/D-TOPO (Invitrogen Corp., Carlsbad, CA) using the TOPO cloning reaction. DNA sequence analysis confirmed that the clone was identical to the original *H. jecorina* QM6a gene sequence. Subsequently, the ORF was transferred to pTrex3g using the LR clonase reaction (Invitrogen) to create the expression vector pTrex3gEndo T with the Endo T ORF flanked by the *cel7a* promoter and termination sequence.

### Transformation of *H. jecorina* and enzyme production

The *H. jecorina* expression strain GICC20000150 was derived from the *H. jecorina* strain RL-P37 [41] by sequential deletion of the genes encoding the four major secreted cellulases (cel7a, cel6a, cel7b and cel5a). Transformation with pTrex3gEndo T was performed using a Bio-Rad Laboratories, Inc. (Hercules, CA) model PDS-1000/He biolistic particle delivery system according to the manufacturer's instructions. *H. jecorina* transformants were selected on solid medium containing acetamide as the sole nitrogen source. For Endo T production, transformants were cultured in a liquid minimal medium containing lactose as carbon source as described previously [42], except that 100 mM piperazine-N, N-bis (3-propanesulfonic acid) (Calbiochem) was included to maintain the pH at 5.5. Culture supernatants were analyzed by SDS-PAGE under reducing conditions and strains that produced the highest level of a band with apparent molecular weight of approximately 34 kDa were selected for further analysis.

### Enzyme purification

The extracellular medium of a *H. jecorina* Endo T overexpression culture (1.2 liter, 990 mg total protein) was concentrated and dialysed against 5 mM ammonium acetate pH 5 by ultrafiltration using polyether sulfon membranes (NWCO 5 kDa, Millipore) to a final volume of 52 ml. A 6 ml sample (114 mg protein) was loaded on a DEAE-Sepharose FF column (10×1 cm, GE Healthcare) equilibrated with 5 mM ammonium acetate. Protein bound to the column was eluted with a linear gradient of 5 mM to 300 mM ammonium acetate, pH 5 (flow rate 1.0 ml/min). The active fractions were again concentrated by ultrafiltration to 4 ml (66 mg) and analyzed with SDS-PAGE. At this stage three species were revealed with a major protein of 33 kDa. A sample was already used for initial screens for crystallization conditions. The rest (33 mg) was further separated to purity with a Biogel P-100 fine column (75×0,75 cm, Biorad) eluted at 0.01 ml/min in 5 mM ammonium acetate pH 5 and concentrated by ultrafiltration.

*H. jecorina* Cel7A was purified from the extracellular medium of both the RL-P37 and the knock-out strain and the catalytic domains were generated by papain digestion as described before [12], [43].

### Protein identification

Mass spectra of purified protein (Endo T and Cel7A core) were acquired on a Q-TOF instrument (Micromass, UK) equipped with a nanospray source. The purified enzyme sample was dissolved in 50% acetonitrile-0.1% formic acid and measured in the positive mode using Protana needles (Odense, UK). Mass spectra were processed using MaxEnt software. Mass accuracy was typically within 0.01–0.02% from the calculated value.

N- and C-terminal sequence analysis of electroblotted samples were performed using a model 476A gas-pulsed liquid phase and a Procise 494C protein sequencer (Applied Biosystems, Foster City, California, USA), respectively [44].

### Protein and activity assays

The concentration of the expressed protein was determined by monitoring the absorbance at 280 nm using a molar absorption coefficient of 47 900 M^−1^ cm^−1^ and a molecular weight of 31.7 kDa. The ENGase activity was monitored using RNase B (Sigma) as substrate. Band shift analysis on SDS polyacrylamide gel was indicative of deglycosylating activity [29]. 10 μl enzyme fractions were incubated with 10 μl RNase B (10 mg/ml dissolved in 100 mM sodium acetate buffer pH 5). Overnight reaction mixtures incubated at 37°C were analyzed using a 15% homogeneous polyacrylamide gel.

### Protein crystallization and data collection

Initial screens for crystallization conditions for Endo T were carried out by the vapor diffusion crystallization technique in hanging drops, using a Greiner 96 well plate and using the Core 96-JCSG+ screen (Qiagen), at 20°C. The crystallization drops were prepared by mixing protein solution containing 16 mg/ml of Endo T with an equal volume of crystallization solution. The protein was crystallized in a solution containing 10% PEG 3350, 0.2 M zinc acetate, and 0.1 M sodium acetate, pH 5.0 at 20°C. Prior to data collection, crystals were flash-frozen in liquid N_2_ using the crystallization solution with 35% PEG 3350, and 30% m-PEG 2000 added as cryo-protectant. The presence of zinc was confirmed by performing an energy scan at the synchrotron beam line on the Endo-T crystals, and measuring the fluorescence emitted by the metal atoms bound in the crystal. Subsequently, the optimal energies and corresponding wavelengths for a MAD data set were determined by fluorescence scanning to maximize the anomalous signal from the bound zinc atoms. A three-wavelengths MAD data set, using zinc as the anomalous scatterer, at wavelengths of 1.28101 Å, 1.28199 Å and 1.27200 Å for the peak, inflection, and remote, respectively, was collected to a resolution of 2.15 Å for all three data sets at the MAD beam line I911-3 at the Swedish synchrotron source MAX-lab, Lund, Sweden. A total of 180 consecutive diffraction images were collected at each wavelength, which resulted in a data completeness of 100%, and redundancy greater than four for each of the three data sets. Subsequently, a high-resolution native data set, 1.3 Å, was collected from a different Endo T crystal. All X-ray diffraction data were processed using the X-ray data integration program Mosflm [45]. The integrated data were scaled using the scaling program Scala in the CCP4i program package [46]. The Endo T crystals were found to belong to the monoclinic space group P21, with approximate unit-cell parameters of: a = 35.4 Å, b = 63.9 Å, c = 59.4 Å, and a β angle of 101.0°. The Matthews coefficient [47] was calculated to be 2.15 Å^3^/Da for one estimated molecule in the asymmetric unit. Further details of data collection and processing are presented in Table 1.

### Structure determination and refinement

Multiple Anomalous Dispersion technique was used for structure determination. The PHENIX program package was used to solve the Endo T structure. Using the program HYSS [48] and the AutoSol Wizard in the PHENIX [49] program package, the positions of seven zinc atoms were readily found, with a figure of merit of 0.52. Using these substructures, Resolve [50] was able to calculated initial phases, perform density modification and build most of the Endo T structure model. The 1.3 Å high-resolution native Endo T dataset was subsequently introduced, and automated model building was carried out by the AutoBuild wizard in PHENIX using the obtained set of phases. The AutoBuild wizard was able to build more than 90% of the initial Endo T structure model, including the solvent model, to 1.3 Å resolution with a final R-factor of 0.28.

After initial structure model building using the auto-building function PHENIX, all further structure refinements were performed using the refinement program REFMAC5 [51]. For cross-validation and R and R_free_ calculations, 5% of the data was excluded from the refinement [52]. Additional water molecules were added using the water picking function in ARP/WARP program package [53]. Throughout the building and refinement of the structure model, the maximum likelihood/_σA_ weighted 2F_obs_−F_calc_ electron density maps [24] were inspected, and the models manually built and adjusted in Coot [54]. Statistics for the final Endo T structure model are shown in Table 2. Figures were prepared with the program PyMol [55]. The coordinates for the final structure model has been deposited at the Protein Data Bank (PDB) [56].

### Sequence alignments and phylogenetic analysis

Protein sequences were aligned using the CLUSTALW algorithm and MACVECTOR 12.5.0 sequence analysis software using default parameters. A phylogenetic tree of the Endo T sequence and its orthologous proteins retrieved with a BLAST search were constructed. At first, 100 sequences were included. However, some gene products from different strains of the same organism were excluded from the final phylogenetic tree. The fungal chitinases Chi18–12 to Chi18–17 were included as an outgroup to root the tree. The phylogenetic tree was constructed by neighbour joining with uncorrected p-values.

## Supporting Information

Figure S1 **An anomalous difference Fourier map, shown as a blue mesh, contoured at 9σ level in a 8** **Å radius region around the zinc ion found bound in the active site of** ***H jecorina*** **Endo T, providing a positive identification for the chemical nature of the bound metal.** Selected protein residues surrounding the zinc atom bound in the catalytic centre are shown as sticks, zinc (modelled in double confirmations) is drawn as grey spheres, selected water molecules surrounding bound zinc are drawn as smaller red spheres.
(EPS)
